# Classification of indoor-outdoor location using combined global positioning system (GPS) and temperature data for personal exposure assessment

**DOI:** 10.1186/s12199-017-0637-4

**Published:** 2017-04-04

**Authors:** B. Lee, C. Lim, K. Lee

**Affiliations:** Department of Environmental Health science, Graduate School of Public Health, Seoul National University, 1 Gwanak-ro Gwanak-gu, Seoul, 08826 Republic of Korea

**Keywords:** Personal exposure, GPS, Time activity, Exposure assessment, Microenvironment

## Abstract

**Objectives:**

The objectives of this study was to determine the accuracy of indoor-outdoor classification based on GPS and temperature data in three different seasons.

**Methods:**

In the present study, a global positioning system (GPS) was used alongside temperature data collected in the field by a technician who visited 53 different indoor locations during summer, autumn and winter. The indoor-outdoor location was determined by GPS data alone, and in combination with temperature data.

**Results:**

Determination of location by the GPS signal alone, based on the loss of GPS signal and using the used number of satellites (NSAT) signal factor, simple percentage agreements of 73.6 ± 2.9%, 72.9 ± 3.4%, and 72.1 ± 3.1% were obtained for summer, autumn, and winter, respectively. However, when temperature and GPS data were combined, simple percentage agreements were significantly improved (87.9 ± 3.3%, 84.1 ± 2.8%, and 86.3 ± 3.1%, respectively). A temperature criterion for indoor-outdoor determination of ~ Δ 2°C for 2 min could be applied during all three seasons.

**Conclusion:**

The results showed that combining GPS and temperature data improved the accuracy of indoor-outdoor determination.

## Introduction

Personal exposure is a factor of both the concentration in the microenvironment, and the duration of the exposure period. The elapsed time in a given place, measures as time-location data, is therefore very useful for determining personal exposure [1, 2]. and accurate determination of the time spent by an individual in the microenvironment is essential for assessing their exposure to air pollution. Several traditional methods have been used for collecting time-location information, including questionnaires, observations, recall interviews and time-activity diaries (TAD) recorded by study participants [3–6]. However, these methods are highly susceptible to variation in the accuracy of recall, and the reliability and compliance of the individuals questioned. Such subjective methods are not ideal for accurately determining individual time-location data, and the development of more reliable methods is needed.

New techniques have been described for the collection of time-location data. The use of a portable global positioning system (GPS) signal receivers has been used to track personal time-location and traveling pattern data, both with and without corresponding manually written TAD [7–9]. This technique offers many advantages over traditional methods, including near-continuous location tracking, high temporal resolution, and minimum reporting burden for participants [10]. However, GPS devices are subject to errors caused by satellite or receiver issues, atmospheric and ionospheric disturbances, multipath signal reflection, and signal loss or blocking [11, 12]. Therefore, it is necessary to develop ancillary methods that support GPS-based methods to enhance the accuracy of time-location information.

In addition to GPS, temperature has been used to classify personal time-location data [13]. Specifically, indoor-outdoor information was collected from elementary school students using a GPS signal receiver and thermometer, and it was possible to detect difference in temperature between indoor and outdoor locations in Montreal, Canada, during winter. Whether this approach could be generally applicable for classifying time-location data elsewhere requires further research. The purpose of the present study was to determine the accuracy of indoor-outdoor classification based on GPS and temperature data in three different seasons.

## Material and methods

A field technician used a GPS signal receiver (GPS 742, Ascen Industry, Korea) and a temperature data logger (UX-100-003, Onset Computer Corporation, Cape Cod, MA, USA) to record data while visiting 53 types of indoor locations that were selected based on the time activity patterns of 2358 individuals in Seoul, Korea [14]. The time activity patterns were measured according to the given time and location which is designed by researchers. For each indoor location, three specific locations (microenvironments) were selected, making a total of 159 indoor environments. Measurements were repeated in three seasons from July to December 2015.

Measurements were performed according to the following protocol: the first 15 min were spent walking outdoors, the next 15 min were spent indoors, and the following 15 min were then spent walking outdoors. A total of 45 min were therefore measured for each indoor location. GPS and temperature data were integrated in 1 min intervals before subsequent analysis. Data between 1 and 15 min were assigned as outdoors, between 16 and 30 min as indoors, and between 31 and 45 min as outdoors, based on TAD data. The time from TAD was synchronized with the time from GPS and temperature data.

The indoor-outdoor location was determined by analysing two GPS signal factors: (1) the presence of data, and (2) the number of satellites (NSAT) used [15]. TAD data were used as the true value to verify the accuracy of GPS signal data-based indoor-outdoor classification. To quantify the accuracy of the GPS signal data analysis, TAD data and GPS signal data were matched every minutes. The simple percentage agreements and Kappa coefficient between GPS-based classification and manually recorded TAD data were calculated.

Temperature criteria used to determine indoor-outdoor location were assessed by comparison with TAD data for three seasons. Temperature criteria were set according to (1) the temperature difference between indoors and outdoors, and (2) the time taken for stabilization of the temperature transition between indoors and outdoors. The sensitivity and specificity for all possible temperature criteria were calculated, and the combination resulting in the highest balanced accuracy was selected as a definitive temperature criterion for the corresponding season. The balanced accuracy is defined as the arithmetic mean of sensitivity and specificity [16].

The accuracy of indoor-outdoor classification following incorporation of temperature data was assessed. To determine the accuracy improvement, two calculations were performed, (1) the first using only GPS signal data, and (2) the second using both GPS signal and temperature data. When GPS signal and temperature data were combined, temperature criteria were applied to consecutive daily data for each season (14, 13, and 16 consecutive days in summer, autumn, and winter, respectively). The specific process was as follows: (1) initial indoor-outdoor assignment using GPS signal data, (2) temperature criteria application, and (3) reclassification. When applying the temperature criteria, the transition between indoors and outdoors was determined, and during the reclassification process, the indoor-outdoor transition was modified. If there were disagreements between the GPS-based transition and the temperature-based transition, temperature-based transition data were considered correct and used in subsequent calculations. The indoor-outdoor classification was then compared with manually written TAD data. To quantify the accuracy of the indoor-outdoor classification for each combination, a simple percentage agreement (the proportion of cases that were identically predicted) and the Cohen Kappa coefficient was calculated. Improvements in the accuracy compared with the classification using the GPS signal alone were then determined.

## Results

The average values of the simple percentage agreements between GPS-based classification and manually recorded TAD data were 73.6 ± 2.9%, 72.9 ± 3.4%, and 72.1 ± 3.1% in summer, autumn, and winter, respectively, and the corresponding average Kappa coefficient values were 0.57 ± 0.04, 0.54 ± 0.03, and 0.53 ± 0.04. There were no significant differences in calculated simple percentage agreements and Kappa coefficients among three seasons. Table 1 shows the balanced accuracy values of the seasonal temperature criteria. In all three seasons, ~Δ 2°C for 2 min was designated as the definitive temperature criterion. The sensitivity and specificity were 0.89 and 0.92, 0.83 and 0.87, and 0.90 and 0.92 for summer, autumn, and winter, respectively, and the corresponding balanced accuracy values were 0.91, 0.86, and 0.91 (Table 1).

**Table 1:** Calculated balanced accuracy values of temperature criteria during the three seasons

Season	Lapsed time (min)	Temperature transition (Δ°C)			
		1	2	3	4
Summer	1	0.64	0.58	0.55	0.52
	2	0.84	0.91	0.71	0.59
	3	0.78	0.88	0.80	0.66
	4	0.74	0.84	0.83	0.77
Autumn	1	0.60	0.54	0.51	0.50
	2	0.79	0.86	0.67	0.58
	3	0.74	0.81	0.76	0.62
	4	0.72	0.79	0.82	0.70
Winter	1	0.59	0.58	0.56	0.54
	2	0.83	0.91	0.71	0.61
	3	0.76	0.86	0.81	0.67
	4	0.74	0.82	0.76	0.77

**Fig. 1:**
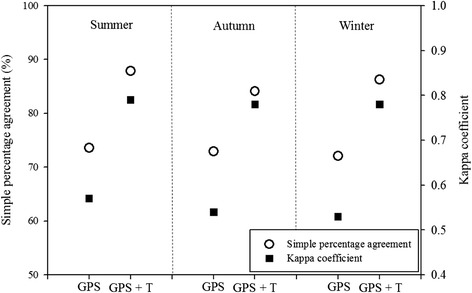
Average simple percentage agreements and Kappa coefficients of indoor-outdoor classification for two different data combinations (T = temperature). The two different data were connected with line. First circle/square in each season means GPS data only, and second circle/square means GPS and temperature data

To determine any improvements in the indoor-outdoor classification, simple percentage agreements and Kappa coefficients were calculated for two classification schemes: (1) the first using only GPS data, and (2) the second using both GPS and temperature data. Figure 1 shows the average simple percentage agreements and Kappa coefficients of indoor-outdoor classifications using these classification schemes for all three seasons. When only GPS data were used, the average simple percentage agreement was 73.6 ± 2.9%, 72.9 ± 3.4% and 72.1 ± 3.1 in summer, autumn and winter. The average Kappa coefficient was 0.57 ± 0.04, 0.54 ± 0.03 and 0.53 ± 0.04 in summer, autumn and winter. When combined GPS and temperature data were used, the average simple percentage agreements were 87.9 ± 3.3%, 84.1 ± 2.8%, and 86.3 ± 3.1% for summer, autumn, and winter, respectively, and the corresponding average Kappa coefficients were 0.79 ± 0.03, 0.78 ± 0.03, and 0.78 ± 0.04. There were no significant differences in the simple percentage agreements and Kappa coefficients among the three seasons.

## Discussion

The values compare with a simple percentage agreement of 90.9 ± 4.6% reported in a previous study [15]. This apparent discrepancy could be due to differences in the measurement methods. In the present study, measurements were performed following a fixed scenario, whereas in the study by Kim et al. [15], simulated activity patterns of individuals were used. In the previous study, the field technician remained in a residential location or other indoor location for 14.9 ± 3.7 and 6.7 ± 3.3 h, respectively, whereas the duration at indoor locations was fixed at 15 min in the present study. Because the duration within an indoor environment was significantly longer in the previous study, the simple percentage agreements were predictably higher than in the present study.

The results of the present study represent a significant advance in our knowledge, although some limitations should be borne in mind. All temperature measurements were performed during the daytime (from 09:00 to 17:00), and because outdoor temperatures decrease continuously after sunset, it is uncertain whether the Δ 2°C for 2 min relationship could be applied to night time as well as daytime, and further experiments are therefore needed.

Although a temperature criterion of Δ 2°C for 2 min could be applied for summer, autumn and winter in the present study, it is unknown whether this could be applied more generally, since this may not be applicable for the entire season in other regions at higher or lower latitudes than Korea. For example, in regions at higher latitude than Korea, the temperature difference between indoors and outdoors is likely to be greater than in Korea during winter, and smaller in summer, and the reverse is likely to be true for regions at lower latitude than Korea. Therefore, additional temperature measurements at higher and lower latitudes are required to verify if Δ 2°C for 2 min is generally applicable.

In another study in Canada, data points were designated as part of the transition state when the temperature changed by more than 0.1°C per min and where the temperature was relatively constant or changed very slowly outside this range [13]. In this previous study, difference between indoor and outdoor temperatures were more than 10°C, compared with the largest difference of 7.8 ± 2.5°C in the winter in the present study. This variation could be due to methodological differences between the two studies. The former study conducted measurements in only two indoor environments; a residential location and an elementary school, whereas the present study conducted measurements in 159 indoor environments. During winter, the elementary school operated a combined heating, ventilation and air conditioning (HVAC) system, and the indoor temperature was therefore relatively constant. However, in the present study, different HVAC systems were used in the 159 indoor environments, and the temperature variation at indoor locations was therefore much larger, and where transitions between indoors and outdoors occurred, the accompanying temperature changes were less than those in the previous study.

Another limitation in applying the temperature criteria more may result from the fixed activity pattern of participants in the present study. Measurements were performed following a highly regimented scenario of 15 min outdoors, followed by 15 min indoors, and a further 15 min outdoors. However, in real world situations, people are unlikely to remain at locations for exactly 15 min. Indeed, the length of stay will be highly variable, and people often remain at indoor environments for less than 15 min. Classifying indoor and outdoor locations through temperature criteria is therefore challenging, and it is necessary to verify the suitability of the temperature criteria for measuring actual daily activity patterns. Additional measurements of activity and temperature made throughout the entire day (i.e. a 24 h time period) are required.

Despite these limitations, the simple percentage agreements and Kappa coefficients, together with the TAD data, showed that the indoor-outdoor classification was improved markedly when temperature data were included. The established temperature criterion could be applied when the temperature difference between indoors and outdoors was significant. Therefore, combining temperature and GPS signal data can enhance the accuracy of indoor-outdoor classification and replace the traditional TAD recording methods of personal exposure to air pollutants.

## Conclusions

Indoor-outdoor locations were classified based on GPS signal and temperature data. Temperature criteria were determined by field measurements at 53 indoor locations during three different seasons. A temperature criterion for the indoor and outdoor transition of Δ 2°C for 2 min could be applied for all three seasons. Combining GPS and temperature data led to a significant improvement in indoor-outdoor classification compared with classification based on GPS data alone.

## Abbreviations

GPS: Global positioning system
HVAC: Ventilation and air conditioning
NSAT: Number of satellites
T: Temperature
TAD: Time-activity diaries
